# Rapid Detection of High-Level Tigecycline Resistance in Tet(X)-Producing *Escherichia coli* and *Acinetobacter* spp. Based on MALDI-TOF MS

**DOI:** 10.3389/fcimb.2020.583341

**Published:** 2020-09-25

**Authors:** Ze-Hua Cui, Zi-Jian Zheng, Tian Tang, Zi-Xing Zhong, Chao-Yue Cui, Xin-Lei Lian, Liang-Xing Fang, Qian He, Xi-Ran Wang, Chong Chen, Bing He, Min-Ge Wang, Ya-Hong Liu, Xiao-Ping Liao, Jian Sun

**Affiliations:** ^1^National Risk Assessment Laboratory for Antimicrobial Resistance of Animal Original Bacteria, South China Agricultural University, Guangzhou, China; ^2^Guangdong Provincial Key Laboratory of Veterinary Pharmaceutics Development and Safety Evaluation, South China Agricultural University, Guangzhou, China; ^3^Guangdong Laboratory for Lingnan Modern Agriculture, Guangzhou, China

**Keywords:** rapid detection, MALDI TOF MS, Tet(X), plasmid-mediated, high-level tigecycline resistance

## Abstract

The emergence and spread of the novel mobile Tet(X) tetracycline destructases confer high-level tigecycline and eravacycline resistance in *Escherichia coli* and *Acinetobacter* spp. and pose serious threats to human and animal health. Therefore, a rapid and robust Tet(X) detection assay was urgently needed to monitor the dissemination of tigecycline resistance. We developed a rapid and simple assay to detect Tet(X) producers in Gram-negative bacteria based on matrix-assisted laser desorption ionization–time of flight mass spectrometry (MALDI-TOF MS). This MALDI^Tet(X)^ test was based on the inactivation of tigecycline by a Tet(X)-producing strain after a 3-h incubation of bacterial cultures with tigecycline. Culture supernatants were analyzed using MALDI-TOF MS to identify peaks corresponding to tigecycline (586 ± 0.2 m/z) and a tigecycline metabolite (602 ± 0.2 m/z). The results were calculated using the MS ratio [metabolite/(metabolite + tigecycline)]. The sensitivity of the MALDI^Tet(X)^ test with all 216 test strains was 99.19%, and specificity was 100%. The test can be completed within 3 h. Overall, the MALDI^Tet(X)^ test is an accurate, rapid, cost-effective method for the detection of Tet(X)-producing *E. coli* and *Acinetobacter* spp. by determining the unique peak of an oxygen-modified derivative of tigecycline.

## Introduction

Tigecycline is a glycylcycline antibiotic and a last resort for treating serious infections caused by multidrug-resistant (MDR) Gram-negative bacteria and even for extensively drug-resistant Enterobacteriaceae and *Acinetobacter* spp. (Doan et al., 2006). Sporadic cases of tigecycline resistance in recent clinical MDR isolates have been associated with either ribosomal protection or high-expression antibiotic efflux mechanisms (Linkevicius et al., 2016). These types of resistance affect antibiotic uptake and target interactions and do not affect the concentration or activity of tigecycline. In addition, these types of resistance can only be transferred vertically and not horizontally (Forsberg et al., 2015).

The appearance of the novel mobile Tet(X) tetracycline destructases has altered this situation with tigecycline and the new glycylcyclines, and Tet(X) activity renders these frontline drugs ineffective. The Tet(X) proteins are flavin monooxygenases that catalyze the degradation of tetracyclines and derivatives (Park et al., 2017). There are currently five that have been discovered in different bacterial species, Tet (X3), (X4), (X5), (X6), and (X7), and all confer high-level resistance to all tetracyclines including tigecycline and the newly Food and Drug Administration (FDA)-approved omadacycline and eravacycline; Tet (X), (X1), and (X2) mediate only first-generation and second-generation tetracycline resistance (He et al., 2019; Sun et al., 2019; Wang et al., 2019; Gasparrini et al., 2020). This poses a great threat to the clinical efficacy of the whole family of tetracycline antibiotics (Fang et al., 2020). Therefore, a rapid and robust Tet(X) detection assay is urgently needed to monitor the dissemination of tigecycline resistance.

Following the discovery of Tet(X3/4) in *Escherichia coli* and *Acinetobacter baumannii*, rapid detection methods based on multiplex real-time PCR were developed that could distinguish between *Tet*(X) variants (Ji et al., 2019; Fu et al., 2020). During this period, our laboratory developed a rapid detection method based on microbial growth tetracycline inactivation method (TIM) that could rapidly detect plasmid-mediated high-level tigecycline resistance. However, TIM required 6.5 h that included 3.5 h for the bacterial growth phase (Cui et al., 2020b). To our knowledge, there is currently no detection method for high-level tigecycline resistance bacteria using matrix-assisted laser desorption ionization–time of flight mass spectrometry (MALDl-TOF MS). Herein, we describe a method for rapid detection of Tet(X)-producing *E. coli* and *Acinetobacter* spp. using MALDI-TOF MS that we have named the MALDI^Tet(X)^ test.

## Materials and Methods

### Bacterial Strains

The 221 strains used in this study were isolated between June 2016 and November 2018 as previously described and had been stored in our archived collection (Chen et al., 2019; Sun et al., 2019; Cui et al., 2020a,b). These included 124 Tet(X)-producing *E. coli* and *Acinetobacter* spp. and 92 non-Tet(X) producers. The group included the five *E. coli* JM109 control strains pBAD24, pBAD24-tet(X3), pBAD24-tet(X4), pBAD24-tet(X6), and American Type Culture Collection (ATCC) 25922. The 124 Tet(X) producers consisted of 38 *tet*(X3) *Acinetobacter* spp., 69 *tet*(X4) *E. coli*, one *tet*(X2)-*tet*(X6) *Acinetobacter* spp., and 16 *tet*(X3)-*tet*(X6) *Acinetobacter* spp. There were also 92 Tet(X)-negative strains consisting of 37 *tet*(X)-negative *E. coli* but carrying at least one other tetracycline resistance gene: [19 *tet*(A), five *tet*(B), three *tet*(D), one *tet*(G), one *tet*(M), two *tet*(A)-*tet*(B), two *tet*(B)-*tet*(D), four *tet*(D)-*tet*(M)] as well as 16 *tet*(X)-negative *Salmonella enteritidis* strains that carried at least one other tetracycline resistance gene: [12 *tet*(B), two *tet*(A)-*tet*(B), one *tet*(B)-*tet*(D), one *tet*(B)-*tet*(M)] and 39 *E. coli* that lacked any tetracycline resistance gene. The five control and 154 test strains were used to establish the MALDI^Tet(X)^ test (Table 1), and 62 test strains were used to test its sensitivity and specificity (Table 2). All strains used to establish and validate the MALDI^Tet(X)^ test were randomly selected based on their species and genotype.

**Table 1:** Characteristics of test strains used to establish the MALDI^Tet(X)^ test.

			**MIC**					
**Species**	***n***	**Genes**	**TC(1^**s**^)^**a**^**	**DOX(2^**s**^)^**a**^**	**TGC(3^**s**^)^**a**^**	**ERA(4^**s**^)^**a**^**	**OMA(4^**s**^)^**a**^**	**MS Ratio**
Control strains	5							
*E. coli-*JM109-pBAD24-tet(X3)	1	*tet*(X3)	64	32	4	4	16	0.5 ± 0.09
*E. coli-*JM109-pBAD24-tet(X4)	1	*tet*(X4)	64	16	8	2	16	0.43 ± 0.11
*E. coli-*JM109-pBAD24-tet(X6)	1	*tet*(X6)	256	32	2	2	16	0.18 ± 0.01
*E. coli-*JM109 - pBAD24	1	non-*tet*(X)^b^	2	0.5	0.03	0.008	0.125	0 ± 0
*E. coli* 25922	1	non-*tet*(X)^b^	2	0.5	0.03	0.06	0.25	0 ± 0
**Test strains**								
Tet(X) producers	92							
*Acinetobacter* spp.	30	*tet*(X3)	64–256	1–64	8–64	4–32	8–64	0.03 ± 0.02–0.57 ± 0.14
*E. coli*	51	*tet*(X4)	32–>256	32–128	1–16	1–16	8–64	0.0067 ± 0.0095–0.48 ± 0.09
*Acinetobacter* spp.	1	*tet*(X2)- *tet*(X6)	>256	128	32	4	16	0.23 ± 0.12
*Acinetobacter* spp.	10	*tet*(X3)-*tet*(X6)	128–>256	8–128	32–64	4–16	8–64	0.02 ± 0.02–0.38 ± 0.12
Non-Tet(X) producers	62							
*E. coli*	19	*tet*(A)	4–256	4–256	0.06–1	0.06–2	0.25–4	0 ± 0
*E. coli*	5	*tet*(B)	256–>256	32–>256	0.125–2	0.25–1	2–8	0 ± 0
*E. coli*	1	*tet*(D)	256	32	0.25	0.25	4	0 ± 0
*E. coli*	1	*tet*(B)- *tet*(D)	256	32	0.25	0.25	4	0.0005 ± 0.0008
*S. enteritidis*	8	*tet*(B)	64–256	8–64	0.5–2	0.06–0.5	1–4	0 ± 0
*S. enteritidis*	1	*tet*(A)- *tet*(B)	128	64	0.5	0.06	2	0.0003 ± 0.0005
*S. enteritidis*	1	*tet*(B)-*tet*(D)	256	64	1	0.25	4	0 ± 0
*S. enteritidis*	1	*tet*(B)-*tet*(M)	256	64	1	0.125	2	0 ± 0
*E. coli*	25	non-*tet*(X)^b^	0.5–1	1	0.125–0.5	0.03–0.06	0.25–1	0 ± 0–0.0014 ± 0.0019

*TET, tetracycline; DOX, doxycycline; TGC, tigecycline; ERV, eravacycline; OMA, omadacycline; MIC, minimum inhibitory concentration; MS, mass spectrometry*.

a *The number in parentheses indicates the generation of tetracycline*.

b *Non-tet(X) strains lack all tet genes as well as tet(X)*.

**Table 2:** Characteristics of test strains used for test validation.

			**MIC**					
**Species**	***n***	**Genes**	**TC(1s)^**a**^**	**DOX(2s)^**a**^**	**TGC(3s)^**a**^**	**ERA(4s)^**a**^**	**OMA(4s)^**a**^**	**MS Ratio**
Tet(X) producers	32							
*Acinetobacter* spp.	8	*tet*(X3)	128–>256	8–128	16–64	4–16	4–64	0.0134 ± 0.0038–0.32 ± 0.13
*E. coli*	18	*tet*(X4)	32–>256	32–64	4–32	4–16	16–64	0.0008 ± 0.0011–0.41 ± 0.22
*Acinetobacter* spp.	6	*tet*(X3)-*tet*(X6)	32–>256	8–128	16–32	4–8	4–16	0.0165 ± 0.0045–0.10 ± 0.02
Non-Tet(X) producers	30							
*E. coli*	2	*tet*(D)	256	128	4	1–2	8	0 ± 0
*E. coli*	1	*tet*(G)	256	64	2	0.5	8	0 ± 0
*E. coli*	1	*tet*(M)	128	64	0.125	0.25	2	0 ± 0
*E. coli*	2	*tet*(A)- *tet*(B)	128–256	64	0.03–0.125	0.03–0.06	0.25–0.5	0 ± 0
*E. coli*	1	*tet*(B)-*tet*(D)	>256	>256	4	2	16	0 ± 0
*E. coli*	4	*tet*(D)-*tet*(M)	128–>256	32–64	0.25–0.5	0.25–0.5	2–4	0 ± 0
*S. enteritidis*	4	*tet*(B)	2–128	0.06–64	0.5	0.06–0.125	2–4	0 ± 0
*S. enteritidis*	1	*tet*(A)- *tet*(B)	128	64	1	0.25	2	0.0003 ± 0.0004
*E. coli*	14	non-*tet*(X)^b^	0.5–1	1	0.125–0.25	0.03–0.06	0.5	0 ±-0.0018 ± 0.0025

*TET, tetracycline; DOX, doxycycline; TGC, tigecycline; ERV, eravacycline; OMA, omadacycline; MIC, minimum inhibitory concentration; MS, mass spectrometry*.

a *The number in parentheses indicates the generation of tetracycline*.

b *Non-tet(X) strains lack all tet genes as well as tet(X)*.

These test strains were isolated from feces (195), dust (three), sewage (eight), flower (one), and soil (nine) samples. The fecal samples were collected from chickens, ducks, geese, pigs, and patients at a tertiary hospital in Guangdong (Supplementary Tables 1, 2). All test strains were identified by MALDI-TOF MS (Axima-Assurance-Shimadzu).

### Antibiotic Susceptibility Testing

Antimicrobial susceptibility assays were performed and interpreted according to Clinical and Laboratory Standards Institute (CLSI) guidelines (CLSI, 2018). Tetracycline and doxycycline minimum inhibitory concentrations (MICs) were determined using the agar dilution method, and the microdilution broth method was used for tigecycline, eravacycline, and omadacycline MIC determinations (Cui et al., 2020b). Tigecycline breakpoints for *E. coli* and *Acinetobacter* spp. were interpreted according to the FDA criteria as susceptible (≤ 2 mg/L), intermediate (4 mg/L), and resistant (≥8 mg/L), and eravacycline and omadacycline were uninterpreted with no breakpoint. *E. coli* ATCC 25922 was used as the quality control strain.

### Detection of Tetracycline Resistance Genes

The tetracycline resistance genes *tet*(X3), *tet*(X4), *tet*(X6), *tet*(A), *tet*(B), *tet*(D), *tet*(G), and *tet*(M) were identified using PCR as previously described (Tuckman et al., 2007; He et al., 2019; Liu et al., 2020). In addition, a *tet*(X) universal PCR test was designed to examine the potential presence of *tet*(X) variants except for *tet*(X3), *tet*(X4), and *tet*(X6).

### The MALDI^Tet(X)^ Test

**Figure 1:**
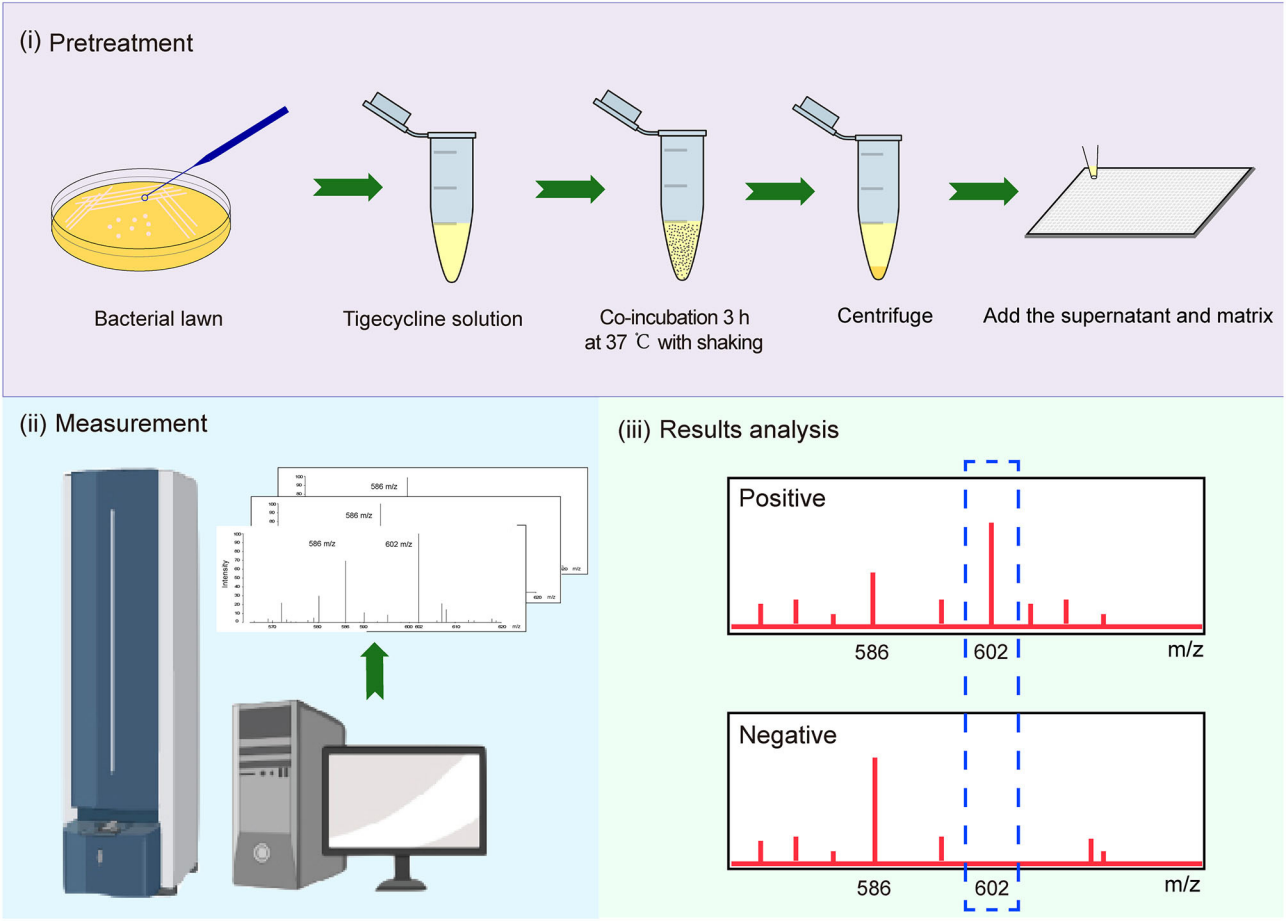
Strategy for identification of Tet(X)-producing *Escherichia coli* (*E. coli*) and *Acinetobacter* spp. using matrix-assisted laser desorption ionization–time of flight mass spectrometry (MALDI-TOF MS).

We used tigecycline as the substrate because Tet (X), (X1), and (X2) mediate tetracycline resistance but not tigecycline resistance (Park et al., 2017; Fang et al., 2020). A 10-μl loopful of an overnight bacterial culture incubated in lysogeny broth agar at 37°C was added to an Eppendorf tube containing 500 μl of 50 mg/L tigecycline (Yuanye, China) and vortexed for 1 min, followed by incubation at 37°C with shaking in the dark for 3 h and was then centrifuged for 3 min at 10,000 × g. A portion (1 μl) of the clear supernatant was spotted onto an MSP 384 target polished steel plate (Shimadzu, Kyoto, Japan) and allowed to dry at room temperature. The matrix (1 μl) α-cyano-4-hydroxycinnamic acid (Sigma-Aldrich, St. Louis, MO, USA) was overlaid onto each target spot. Mass spectra were acquired using a Shimadzu performance mass spectrometer and Shimadzu Biotech MALDI-MS software (Shimadzu) operating in positive linear ion mode between 100 and 1,000 Da. The parameters were set as follows: ion source 1, 20 kV; ion source 2, 2.62 kV; lens, 6 kV; pulsed ion extraction, 114 ns; electronic gain, enhanced; mode, low range; mass range selection, 80–1,120 Da; laser frequency, 60 Hz; digitizer trigger level, 2,500 mV; laser attenuator, 25%; and laser range, 40%. A total of 500 shots were acquired in one position for each spectrum (Figure 1).

*E. coli* strains JM109-pBAD24 and ATCC 25922 served as negative controls, while pBAD24-*tet*(X3), pBAD24-*tet*(X4), and pBAD24-*tet*(X6) cultured in the presence of 0.1% L-arabinose were used as positive controls.

### Spectral Analysis

Spectra were analyzed using Shimadzu Biotech MALDI-MS software (Shimadzu). Peaks for tigecycline (C_29_H_39_N_5_O_8_) (586 ± 0.2 m/z) and its only known metabolite (C_29_H_39_N_5_O_9_) (602 ± 0.2 m/z) were manually labeled, and their intensities noted (Moore et al., 2005). MS ratios of intensities were calculated according to metabolite/(metabolite + tigecycline) [M/(M + T)] and were calculated for the 154 cutoff strains to establish a threshold ratio between Tet(X) producers and non-producers. Strains were classified as Tet(X) producers when this ratio was superior or equal to the cutoff values. All experiments were carried out on three independent bacterial cultures on three different days. MS ratios were calculated as the mean values from three independent experiments.

### Statistical Analysis

Descriptive analyses of MS ratios were performed using functions provided in Excel 2010 (Microsoft). A receiver operating characteristic (ROC) curve analysis was used to determine the optimal cutoff value (Hanley and Mcneil, 1982), and the optimal cutoff point was defined by the Youden index (Youden, 1950). The ratio-based model was validated for the results of 62 well-characterized isolates that had been previously identified using PCR.

## Results

### Antibiotic Susceptibility Testing

Tigecycline MICs for our 221 test strains ranged from 0.03 to 64 mg/L. In the group of strains used to establish the MALDI^Tet(X)^ test, 49/51 *tet*(X4)-positive *E. coli* strains were tigecycline resistant, one was intermediate, and one was tigecycline susceptible. Of the strains used to establish the MALDI^Tet(X)^ test, all the Tet(X)-producing *Acinetobacter* spp. strains were tigecycline resistant, whereas all non-Tet(X) producers were tigecycline susceptible. The strains used for the MALDI^Tet(X)^ test validation included 16/18 of *tet*(X4)-positive *E. coli* strains that were determined tigecycline resistant, one tigecycline intermediate, and one tigecycline susceptible. Of the strains used to validate the MALDI^Tet(X)^ test, all the Tet(X)-producing *Acinetobacter* spp. strains were tigecycline resistant, whereas 28/30 of the non-Tet(X) producers were tigecycline susceptible; the two *E. coli* strains carrying *tet*(D) were tigecycline intermediate (Tables 1, 2).

### Detection of Tet(X) Producers Using the MALDI^Tet(X)^ Test

**Figure 2:**
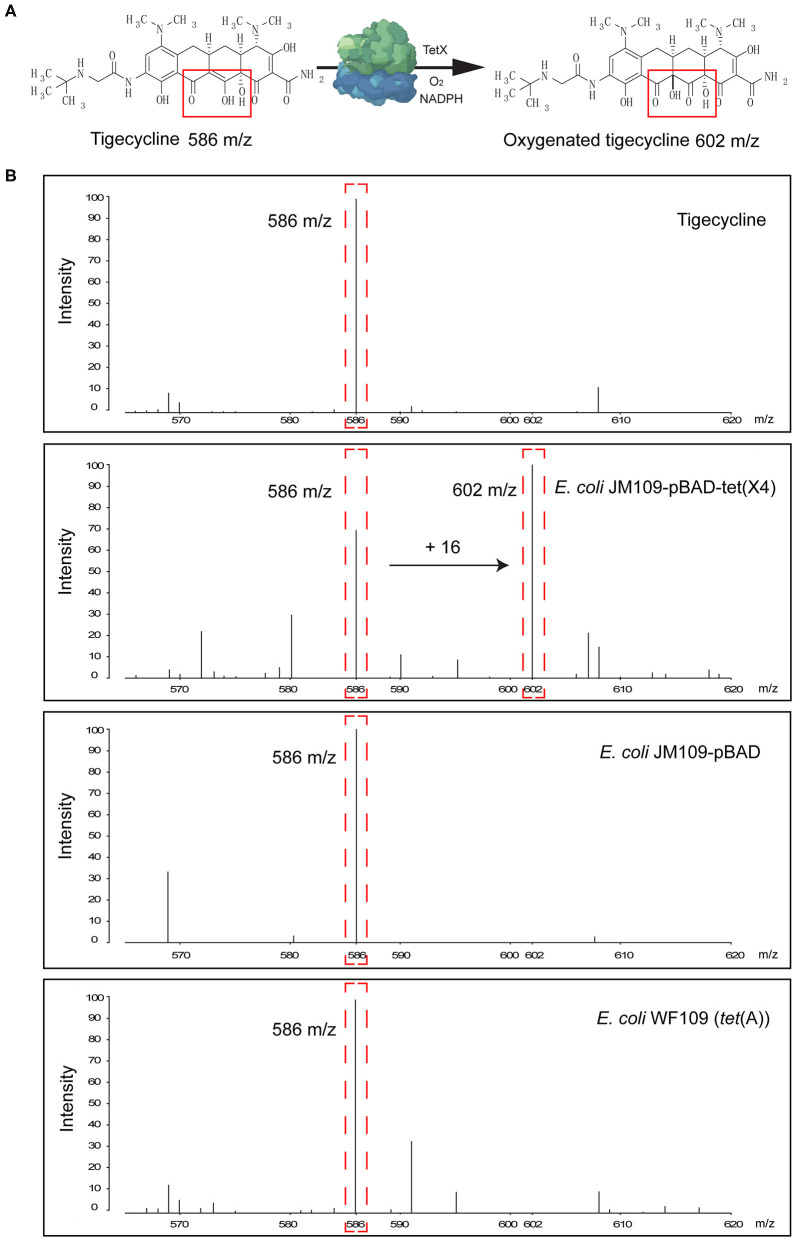
Representative results of the matrix-assisted laser desorption ionization–time of flight mass spectrometry (MALDI-TOF MS) detection of Tet(X) producers and non-producers. **(A)** The structure of tigecycline and the product of the oxygen-modified derivative of tigecycline. Tigecycline and oxygen-modified tigecycline possessed peaks at 586 ± 0.2 and 602 ± 0.2 m/z, respectively. **(B)** Representative MALDI-TOF MS spectra of tigecycline oxygenation assays after a 3-h incubation at 37°C. Peaks of interest are denoted by dashed red lines and represent the tigecycline peak at 586 ± 0.2 m/z and its metabolite at 602 ± 0.2 m/z.

Tigecycline inactivation by Tet(X) occurs *via* covalent modification at C11a of the tetracycline nucleus and results in O addition. The product peak with an m/z of 602 ± 0.2 corresponded to the addition of one oxygen atom to tigecycline (586 ± 0.2 m/z) (Figure 2A) (Moore et al., 2005). As expected, the 602 ± 0.2 m/z peak appeared in the mass spectrogram of the Tet(X)-producing *E. coli* and *Acinetobacter* spp. when the MALDI^Tet(X)^ test was used. This peak was lacking in non-Tet(X) producers (Figure 2B). We corroborated this by the analysis of 154 test strains that included 92 Tet(X) producers and 62 non-producers. The intensities of the peaks corresponding to tigecycline (586 ± 0.2 m/z) and oxygen-modified tigecycline (602 ± 0.2 m/z) were recorded from three independent experiments. The latter peak corresponded to samples taken from Tet(X) producers (Table 1).

The calculation of MS ratios allowed accurate distinction between Tet(X) producers and non-producers. We therefore performed additional tests using the JM109-positive control strains that possessed arabinose-inducible *tet*(X) genes. The MS ratios of pBAD24-*tet*(X3), pBAD24-*te*t(X4), and pBAD24-*tet*(X6) were 0.5 ± 0.09, 0.43 ± 0.11, and 0.18 ± 0.01 m/z, respectively, while the ratios for the two negative controls ATCC 25922 and pBAD24 were 0. Tests of our group of 30 *tet*(X3)-positive *Acinetobacter* spp. included MS ratios that ranged from 0.03 ± 0.02 to 0.57 ± 0.14 and the 51 *tet*(X4)-positive *E. coli* values ranged from 0.0067 ± 0.0095 to 0.48 ± 0.09. However, in three independent experiments, a single isolate in this group had an MS ratio of 0 in two of the experiments. Similarly, 2/11 of the *Acinetobacter* spp. carrying the *tet*(X6) gene presented MS ratios of 0 in 1/3 and 2/3 experiments. The non-Tet(X)-producing *E. coli* and *S. enteritidis* strains had MS ratios of 0 for 57/62 of the samples, and the remaining five strains possessed MS ratios that were not 0 in 1/3 experiments (Supplementary Table 1).

**Figure 3:**
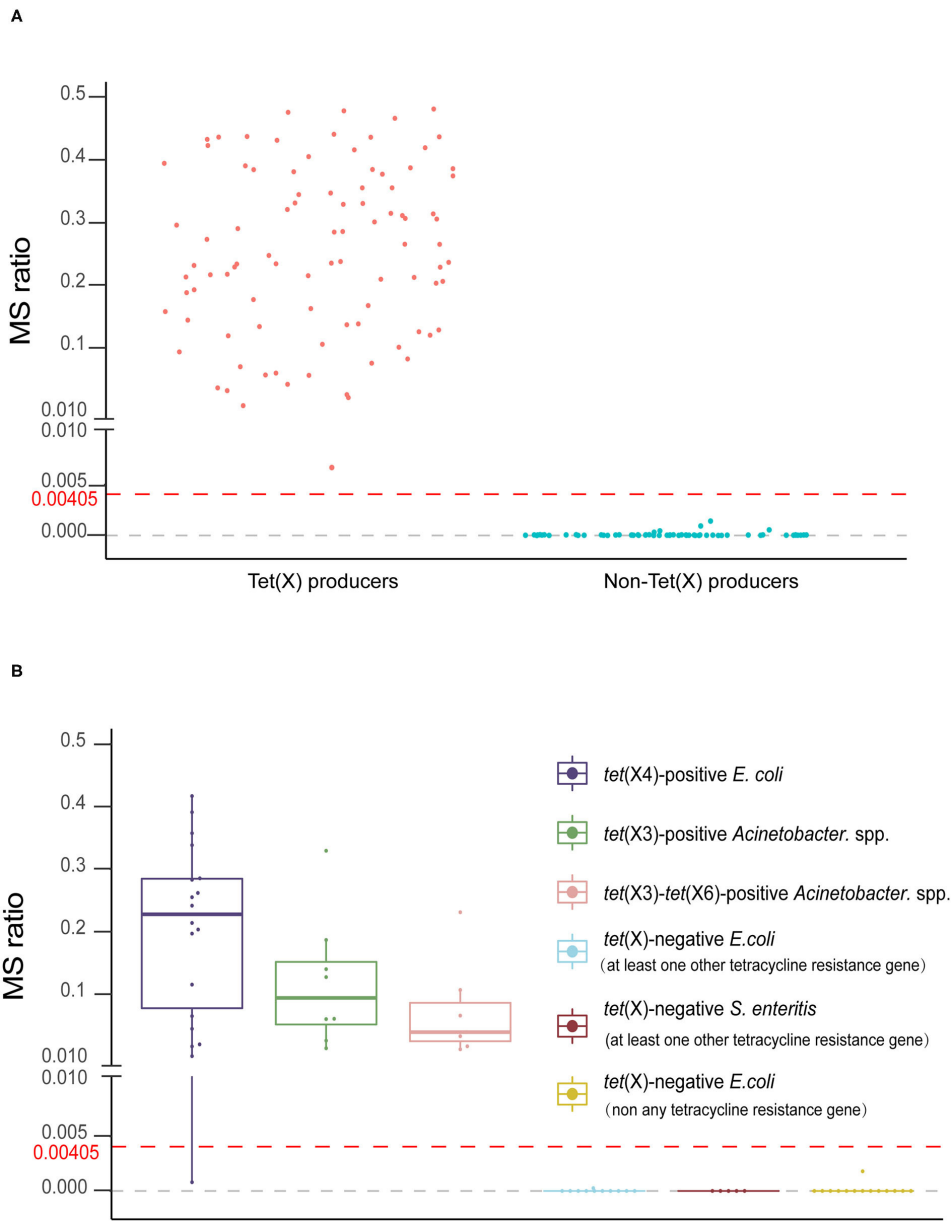
The MALDI^Tet(X)^ test results using test strains. **(A)** Distribution of the mass spectrometry (MS) ratios used to establish the MALDI^Tet(X)^ test. The cutoff value of 0.00405 can clearly distinguish between Tet(X) producers and non-producers **(B)** MS ratio distribution of 32 Tet(X) producers and 30 non-producers used for assay validation. Three independent experiments were performed for each strain.

The MS ratios of Tet(X)-producing strains ranged from 0.0067 ± 0.0095 to 0.57 ± 0.14, whereas in the non-producers, these ratios ranged from 0 to 0.0014 ± 0.0019 (Figure 3A). Receiver operating characteristic (ROC) analysis allowed us to define a cutoff value for the MS ratio at 0.00405 that discriminated Tet(X) producers from non-producers. The latter group displayed MS ratios <0.00405, whereas all Tet(X)-producing strains had MS ratios > 0.00405 (Figure 3B).

### Model Validation

We validated our model using 62 strains that were blind tested using the calculated cutoff of 0.00405. The 32 Tet(X)-producing *E. coli* and *Acinetobacter* spp. possessed MS ratios that ranged from 0.0008 ± 0.0011 to 0.41 ± 0.22, and only a single *tet*(X4)-positive *E. coli* had an MS ratio below the cutoff value in three independent experiments (0, 0, and 0.0024). Four *tet*(X4)-positive *E. coli* and three *tet*(X3)-*tet*(X6) had MS ratios of 0 in some of the replicates. In the 30 non-Tet(X) producers we examined, the MS ratios ranged from 0 ± 0 to 0.0018 ± 0.0025, and these results were completely consistent with the MALDI^Tet(X)^ test results. Similarly, 2/30 of the non-Tet(X)-producing *E. coli* and *S. enteritidis* strains generated MS ratios that were not 0 in 1/3 experiments (Supplementary Table 2).

The group of 62 validation strains yielded only one *tet*(X4)-positive *E. coli* that was incorrect using the MALDI^Tet(X)^ test. The sensitivity and specificity of the MALDI^Tet(X)^ test using the validation group were 96.88 and 100%, respectively, and using all 216 strains, the sensitivity was 99.19% and specificity was 100%.

## Discussion

MALDI-TOF MS is an important method for bacterial identification because it is rapid and reliable and therefore is widely deployed in microbiology laboratories around the world (Pan et al., 2007; Stevenson et al., 2010; Huang et al., 2013). In the present study, we developed a MALDI-TOF MS procedure (the MALDI^Tet(X)^ test) to rapidly detect Tet(X) producers in *E. coli* and *Acinetobacter* spp. within 3 h.

There are numerous genotypic and phenotypic methods currently in use for the detection of Tet(X)-producing *E. coli* and *Acinetobacter* spp. Genotypic detection using PCR allows high sensitivity and specificity, but high-throughput detection cannot be achieved due to the lack of universal primers for each gene subtype (Cavanaugh and Bathrick, 2018; Shahi et al., 2018). Multiplex real-time PCR can simultaneously detect multiple gene subtypes but is unable to identify unknown genes (Hawkins and Guest, 2017; Minkus et al., 2019). Since Tet(X) is a tetracycline degradation enzyme, it can be phenotypically detected using tetracycline degradation assays that can be assessed using liquid chromatography–tandem mass spectrometry (LC-MS/MS), but this requires a complex sample pretreatment process (Ji et al., 2010; Palmer et al., 2010). Agar well-diffusion methods are also in use but are time-consuming, although recent modifications using *Bacillus stearothermophilus* as the indicator and color reagent addition have significantly shortened the time required for detection (Mata et al., 2014; Hussein et al., 2015; Balouiri et al., 2016; Galvão et al., 2016; Wu et al., 2019; Cui et al., 2020b). In contrast, the MALDI^Tet(X)^ test is extremely rapid and simple and requires equipment that are now available in many clinical microbiology laboratories. This study is the first to demonstrate the use of MALDI-TOF MS to detect Tet(X)-producing *E. coli* and *Acinetobacter* spp. MALDI-TOF MS has been used for phenotypic characterization of carbapenemase-producing Enterobacteriaceae (CPE) and colistin-resistant Enterobacteriaceae and relies on the detection of carbapenem hydrolysis products in <30 min because of the high catalytic efficiency of carbapenemases (Lasserre et al., 2015). In a similar manner, MALDI-TOF MS has also been used to determine whether a bacterial strain is colistin resistant by the direct measurement of Lipid A modifications, and the process takes only 15 min (Dortet et al., 2018). Our MALDI^Tet(X)^ test identified Tet(X)-producing *E. coli* and *Acinetobacter* spp. at a rate and efficiency to that of the CPE detection method, and both rely on the metabolite identification. However, Tet(X) enzyme efficiency is much lower than for the carbapenemases, and the overall reaction process requires more time (Queenan and Bush, 2007; Park et al., 2017).

Theoretically, the covalent coupling of oxygen to tigecycline occurs only if the test strain is a Tet(X) producer resulting in a 602 ± 0.2 m/z peak. In the group of 124 Tet(X)-producing strains we used for this study, only eight had MS ratios of 0 in 1/3 or 2/3 experiments; these anomalies were most likely the result of a Tet(X) possessing weak activity, and meanwhile, we did identify 7/92 non-Tet(X) producers that possessed low-intensity 602 ± 0.2-m/z peaks. To ensure that the method has higher sensitivity and specificity, we defined a robust cutoff value for the MS ratio of 0.00405, but when coupled with the presence or absence of a 602 ± 0.2-m/z peak, the accuracy of the MALDI^Tet(X)^ test was still very high. The results of the non-Tet(X) producers indicated that the presence of other *tet* genes that mediate tetracycline resistance will not influence the MALDI^Tet(X)^ test results; these results are reasonable and easy to understand, since tigecycline inactivation by Tet(X) occurs *via* covalent modification at C11a of the tetracycline nucleus.

We examined only bacterial strains that possessed the *tet*(X3), *tet*(X4), and tet(X6) genes, although this method can be extended to examine additional isotypes, and specificity and sensitivity should be reexamined. In theory, the MALDI^Tet(X)^ test can detect Tet(X)-producing strains of any species, which will need to be studied in the future. In addition, our method should be extended for the direct detection of these organisms in blood and urine samples (Meier and Hamprecht, 2019), and further evaluation of the MALDI^Tet(X)^ test is worthy of further study.

## Conclusions

We have developed a MALDI-TOF MS-based assay to identify Tet(X)-producing *E. coli* and *Acinetobacter* spp. by determining a unique peak of an oxygen-modified derivative of tigecycline. The overall manipulations were simple and rapid, and this phenotypic detection method is appropriate as a routine test in clinical microbiology laboratories that have access to the MALDI-TOF MS instrumentation. The test is low cost and possesses excellent sensitivity and specificity.

## Data Availability Statement

All datasets generated for this study are included in the article/Supplementary Material.

## Ethics Statement

Clinical strains isolated from humans in this study were provided by the Third Affiliated Hospital of Sun Yat-sen University. This study was carried out in accordance with the recommendations of ethical guidelines of South China Agricultural University. SCAU Institutional Ethics Committee did not require the study to be reviewed or approved by an ethics committee because we are not involved in the isolation of bacteria.

## Author Contributions

JS, Y-HL, and X-LL designed the study. Z-HC, Z-JZ, TT, Z-XZ, and C-YC carried out the experiments. JS, X-RW, CC, BH, and M-GW analyzed the data. JS, Z-HC, L-XF, and Z-JZ wrote the draft of the manuscript. All authors read and approved the final manuscript. All authors contributed to the article and approved the submitted version.

## Conflict of Interest

The authors declare that the research was conducted in the absence of any commercial or financial relationships that could be construed as a potential conflict of interest.
